# Early Retinal and Choroidal Coat Thickness Changes after Intravitreal Dexamethasone Implant Injection for Diabetic Macular Edema

**DOI:** 10.4274/balkanmedj.2017.1013

**Published:** 2018-09-21

**Authors:** Fatih Horozoğlu, Özkan Sever

**Affiliations:** 1 Department of Ophthalmology, Namık Kemal University School of Medicine, Tekirdağ, Turkey

**Keywords:** Choroidal coat, Dexamethasone, diabetes mellitus, macular edema

## Abstract

**Background:**

Intravitreal steroid injection is one of the treatment options for diabetic macular edema. Dexamethasone implant is the most novel form of intravitreal steroid therapy. Improvement in macular thickness is a well-known effect of Dexamethasone implant however, subfoveal choroidal coat thickness changes require investigation.

**Aims:**

To evaluate the early central macular thickness and subfoveal choroidal thickness alterations after single-dose dexamethasone implant injection in diabetic macular edema.

**Study Design:**

Cross-sectional study.

**Methods:**

We identified 29 patients with diabetic macular edema (29 eyes) who underwent optical coherence tomography and fundus fluorescein angiography. All patients received a single-dose intravitreal Dexamethasone implant and were followed up for central macular thickness and subfoveal choroidal thickness alterations for 1 hour, 1 week, 1 month, and 3 months post-injection.

**Results:**

The preoperative mean central macular thickness and subfoveal choroidal thickness measurements were 592.3±122.3 (412–879) μm and 264.8±53.7 (165–397) μm, respectively. Central macular thickness measurements decreased significantly in the first hour (p<0.050) and continued to decrease until the third month (p<0.001), whereas the subfoveal choroidal thickness decrement was only significant on the first day (p<0.05). Decreases in subfoveal choroidal thickness and central macular thickness of 1.5% and 5% were observed at 1 hour; however, the difference was not significant (p>0.050). The decrease in central macular thickness was significantly greater than that in subfoveal choroidal thickness at 1 day, 1 week, 1 month, and 3 months (p<0.001).

**Conclusion:**

Intravitreal Dexamethasone implant has a meaningful effect on central macular thickness in patients with diabetic macular edema, while subfoveal choroidal thickness decreases significantly at first day.

Diabetic macular edema (DME) is the major cause of vision loss in patients with diabetes (1). Laser photocoagulation, anti-vascular endothelial growth factor (anti-VEGF) injections, corticosteroid injections and pars plana vitrectomy are among the previously used treatment modalities (2,3,4,5). The effects of anti-angiogenesis and decreasing vascular permeability makes VEGF a logical target in the treatment of diabetic macular edema. While anti-VEGF treatment has a positive effect on visual improvement in diabetic macular edema, there have been several limitations to its use, such as the need for multiple injections and, in some patients, anti-VEGF treatment is not successful (6). Fluocinolone (Iluvien^®^, Alimera) and Dexamethasone (Dx) implant (Ozurdex^®^, Allergan) are the approved corticosteroid therapies for the treatment of diabetic macular edema (7,8). Although anti-VEGF treatment improves visual acuity, it requires monthly injections. Dx implant was able improve vision by 4 letters with fewer injections compared with anti-VEGF agents (7). Changes in central macular thickness and subfoveal choroidal thickness before and after drug injection have been of interest since the invention of optical coherence tomography in recent years (9,10). Favorable results were obtained with Dx implant treatment in anti-VEGF-resistant eyes with diabetic macular edema  and there was a correlation between the reductions in central macular thickness  and subfoveal choroidal thickness (11). In this study, we aimed to investigate early choroidal and macular thickness changes after the injection of Dx implant.

## MATERIALS AND METHODS

This cross-sectional study was conducted in accordance with the Declaration of Helsinki on patients with diabetic macular edema who received intravitreal Dx implant (Ozurdex^®^; Allergan, Irvine, California, USA) (7) over the period of May 2014 to June 2016. Best-corrected visual acuity (BCVA) with a Snellen chart, biomicroscopic evaluation with a slit-lamp, intraocular pressure measurement with tonometry, and fundus examination with a 78 D lens were the used as the standard ocular examination for all patients at the first visit. Fundus fluorescein angiography and optical coherence tomography evaluations were performed before treatment.

### Ethics

The Institutional Ethics Committee approved the study protocol. The risks of surgical treatment were explained to all patients, and informed consent was obtained.

### Optical coherence tomography measurements

**Figure 1:**
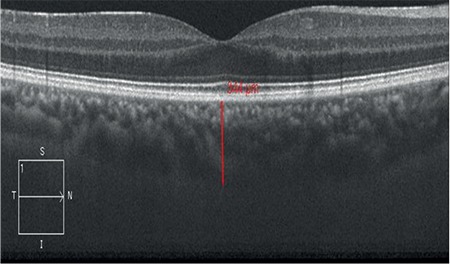
Subfoveal choroidal thickness measurement of a patient who had received intravitreal Dexamethasone implant (Ozurdex^®^).

Macular thickness was measured by spectral domain optical coherence tomography (Cirrus HD^TM^, Carl Zeiss Meditec) with the machine’s own software protocol as the distance between the internal limiting membrane and the retinal pigment epithelium. Raster mode was used to scan the fovea with 1024 A-scans per line. The enhanced depth imaging (EDI-optical coherence tomography) mode of Cirrus HD^TM^ was used for subfoveal choroidal thickness  evaluation between retinal pigment epithelium and the internal scleral border from the thinnest zone corresponding fovea. Manual measurement was performed with a digital caliper by a technician who was blinded to treatment. Subfoveal choroidal thickness measurement of a patient is shown in Figure 1. Macular edema was defined as a central macular thickness ≥300 μm.

### Patients

Eyes that met the following criteria were excluded: 1) vitreous hemorrhage or cataract-like media opacities, 2) foveal avascular zone irregularities detected by Fundus fluorescein angiography , 3) six clock hours or more of macular capillary non-perfusion on Fundus fluorescein angiography , 4) any neovascularizations, 5) history of vitreoretinal surgery, or 6) preoperative vitreomacular interface disease detected by optical coherence tomography. The Dx implant was injected intravitreally to all patients, and patients were examined for central macular thickness and subfoveal choroidal thickness changes 1 hour, 1 day, 1 week, 1 month, and 3 months postoperatively. Following Ozurdex^®^ implantation, measurement of BCVA, slit-lamp biomicroscopy, fundus examination by 78 D lens, tonometry and optical coherence tomography evaluation were repeated at each visit.

### Statistical analysis

The program PASW Statistics 18 was used to analyze the results. Percentage was used for qualitative data and mean ± standard deviation, for quantitative data. Treatment effectiveness was evaluated for each patient by comparing pre- and post-injection subfoveal choroidal thickness and central macular thickness at each visit by paired-sample t-test. Subfoveal choroidal thickness  and central macular thickness groups were compared by independent-sample t-test. Statistical values of p<0.05 were accepted as significant.

The program G power 3.1 was used to calculate the sample size and power. In a choroidal and macular thickness calculation repeated five times with n=29, if the probability of a type 1 error (α)=0.05, the partial Eta squared was 0.212 and 0.795, the correction for non-sphericity e was 0.25 and 0.446, the power was 75.47% and 100%, respectively.

## RESULTS

We identified 29 patients with diabetic macular edema (29 eyes) who underwent single-dose Dx implant injection. Of the 29 patients, 16 were female and 13 were male. Table 1 shows the baseline patient characteristics. The mean number of previous anti-VEGF injections was 1.55 (0 to 6). Preoperative and postoperative central macular thickness and subfoveal choroidal thickness changes are listed in Table 2.

Preoperative mean thickness was 592.3±122.3 (412-879) µm in central macular thickness and 264.8±53.7 (165-397) in subfoveal choroidal thickness. Table 2 shows preoperative and postoperative central macular thickness and subfoveal choroidal thickness measurements. Measurements taken for central macular thickness at 1 hour, 1 day, 1 week, 1 month, and 3 months after Dx implantation. As we compare the change between the preoperative and post-injection values, all of them were significantly decreased (p˂0.050). However, the decrement in subfoveal choroidal thickness from preoperative values was only significant at 1 day postoperatively (p<0.050). Table 3 shows a comparison of the differences in central macular thickness and subfoveal choroidal thickness. Decreases in subfoveal choroidal thickness and central macular thickness of 1.5% and 5% were observed at 1 hour; however, the difference was not significant (p>0.050). The decrease in central macular thickness was significantly greater than that in subfoveal choroidal thickness at 1 day, 1 week, 1 month, and 3 months (p<0.001).

## DISCUSSION

Evaluation of the choroid was of interest previously, and until the advent of EDI-optical coherence tomography, imaging of the choroid was performed by using indocyanine green angiography (ICGA) or laser Doppler flowmetry. Recently reported choroidal vascular abnormalities in diabetic eyes included dilatation, obstruction, non-perfusion, tortuosity increase, and remodeling (12,13,14). It is speculated that diabetic choroidopathy (DC) would have a role in explaining visual loss in diabetic eyes without retinopathy (15). An increase in the resistance index has been reported by laser Doppler flowmetry beneath the fovea and might be a result of progressive reduction of choroidal blood flow and volume in diabetic patients, even in eyes without retinopathy (16). These data implicate DC as a potential trigger of the development of retinopathy. Thus, ICGA and laser Doppler flowmetry can provide valuable data about the choroid, and research has been focused on increasing understanding and identifying other possible roles of the choroid. With the introduction of EDI-optical coherence tomography (17), several studies concentrated on the choroid, and much was learned about retinochoroidal diseases (18,19). Tracking changes in choroidal thickness in patients with diabetic macular edema might be useful to predict the effect of anti-VEGF treatment (20). Although there are many studies evaluating subfoveal choroidal thickness as well as choroidal area in diabetic patients, there is still controversy surrounding choroidal thickness in diabetic eyes. Regatieri et al. (21) reported that patients with diabetic macular edema have thinner choroid than non-diabetic patients, whereas Kim et al. (22) revealed that subfoveal choroidal thickness increased with increasing severity of diabetic retinopathy (from no diabetic retinopathy to proliferative diabetic retinopathy) and in the presence of diabetic macular edema, especially in eyes with serous retinal detachment.

In the current study, after single-dose injection of Dx implant, subfoveal choroidal thickness decreased significantly on the first day; however, this decrease was not maintained at 1 and 3 months follow-up in eyes with diabetic macular edema. However, central macular thickness decreased beginning from the first hour after injection and continue to decrease until 3 months follow-up. Previously, mean subfoveal choroidal thickness was reported to be 280 μm in a healthy Turkish population with a mean age of 47 years (23). They reported a decrease in subfoveal choroidal thickness of 3.14 μm for each year of age. We found a mean subfoveal choroidal thickness of 264 μm in a diabetic population with a mean age of 58 years in this study. As we compare the mean subfoveal choroidal thicknesses of population between Ozdogan Erkul et al. (23) study and our study, there is a slight decrease (16 µm) in our diabetic population that might be the negative effect of previous panretinal photocoagulation. The difference between the healthy population and our diabetic population might have been caused by previous panretinal photocoagulation in some eyes, which is known to decrease subfoveal choroidal thickness (24), and the older age of our patients.

A prospective study that compared the effects of triamcinolone acetonide and bevacizumab injections on choroidal thickness in diabetic macular edema showed a significant decrease in subfoveal choroidal thickness from 24 hours to 12 weeks with triamcinolone acetonide (25). In a similar study that demonstrated changes in choroidal thickness after the injection of Dx implant in eyes with diabetic macular edema and a history of multiple injections of anti-VEGF, the decreases in subfoveal choroidal thickness and central macular thickness were similar and were significant at 1 and 3 months postoperatively (11). In this study, mean preoperative choroidal thickness was 288 microns and decreased to 260 and 266 microns after 1 and 3 months, respectively. The authors mentioned that previous anti-VEGF injections before Dx injection might have impacted on choroidal thickness measurements. As our patients were not fully included with refractory diabetic macular edema and persistent fluid despite anti-VEGF injections, this may also be a limitation of our study. We excluded eyes with PDR and active neovascularization from our study. This group of eyes is reported to have the thickest subfoveal choroidal thickness among eyes with diabetic retinopathy (22). This may explain the discrepancy between our results and those of Kim et al. (11).

Central macular thickness  was decreased after injection of the Dx implant at the postoperative 1^st^ hour, and this decrease was maintained for 3 months. This is a well-known effect of Dx implant in diabetic macular edema, even in eyes with persistent fluid despite multiple anti-VEGF injections (11,26,27,28,29). Our study confirmed these results.

Manual measurement of subfoveal choroidal thickness, heterogeneous groups of patients with and without previous panretinal photocoagulation, and the variable timing of previous anti-VEGF injections were the limitations of our study. Although eyes were not divided into groups corresponding retinopathy stages, eyes with active neovascularization were not included in the study. Despite these limitations, our study is the first to report early retinal and choroidal thickness changes as well as their correlations in diabetic patients who received Dx implant.

In conclusion, this study concentrated on early effects of Dx implant on subfoveal choroidal thickness and compared the changes in subfoveal choroidal thickness and central macular thickness. Dx implant is found to be effective at decreasing macular edema in diabetic eyes for up to 3 months. However, subfoveal choroidal thickness  exhibited a significant decrease only on the first day after Dx implant injection. A comparison of subfoveal choroidal thickness  and central macular thickness changes did not reveal a significant difference. The effect of Dx implant on choroidal thickness still is uncertain. Further studies enabling non-invasive visualization of vascular structures with optical coherence tomography angiography and other techniques will identify the effects of injections on the retina and choroid in diabetic macular edema.

## Figures and Tables

**Table 1:**
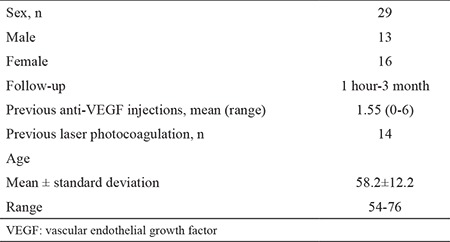
Patient characteristics

**Table 2:**
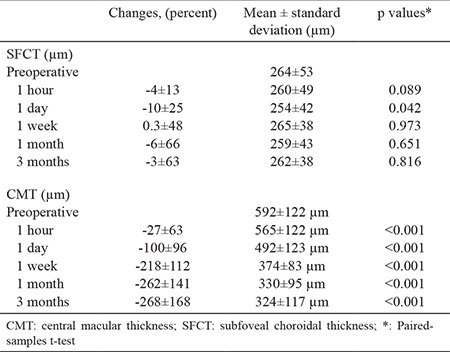
Central macular thickness and subfoveal choroidal thickness changes after single-dose intravitreal Dexamethasone implant

**Table 3:**
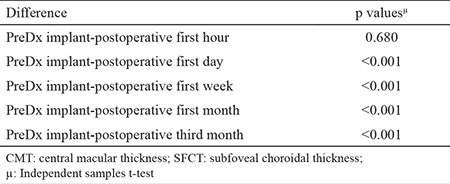
Comparison of subfoveal choroidal thickness and central macular thickness changes in eyes after single-dose intravitreal Dexamethasone implant
